# The Correlation between Lung Ultrasound and Pathology in Rat Model of Monocrotaline-Induced Pulmonary Hypertension

**DOI:** 10.1155/2024/6619471

**Published:** 2024-07-23

**Authors:** Yan-Fen Zhong, Bin-Bin Liang, Xiao-Feng Zhang, Ji-Wu

**Affiliations:** Department of Ultrasonic Medicine The First Affiliated Hospital of Guangxi Medical University, No. 6 Shuangyong Road, Nanning 530021, China

## Abstract

Pulmonary hypertension (PH) is a progressive and complex pulmonary vascular disease with poor prognosis. The aim of this study was to provide a new understanding of the lung pathology of disease and a noninvasive method in monitoring the establishment of animal models for basic and clinical studies of PH, indeed to explore clinical application value of lung ultrasound for patients with PH. Totally 32 male SD rats were randomly divided into control group, MCT (monocrotaline) group, PDTC (pyrrolidine dithiocarbamate) group, and NS (normal saline) group. Rats in the MCT group, PDTC group, and NS group received single intraperitoneal injection of MCT, while the control group received the same dose of NS. Then, PDTC group and NS group received PDTC and NS daily for treatment at the end of the model. Each group received lung ultrasound examination and measurement of pulmonary arterial pressure (PAP). Then, the rats were sacrificed to take the lung specimens to being observed. The ultrasound and pathological results were analyzed with a semiquantitative score. With the pulmonary artery pressure increases, the MCT group had a higher pulmonary ultrasound score and pathological score compared with the control group (*p* < 0.05). After PDTC treatment, the pulmonary ultrasound score and the pathological score decline (*p* < 0.05). We investigated both lung ultrasound scores, and the pathological scores were positively correlated with mean pulmonary artery pressure (mPAP) (both *r* > 0.8, *p* < 0.0001). Moreover, lung ultrasound scores were positively correlated with pathological scores (*r* > 0.8, *p* < 0.0001). We elucidated lung ultrasound evaluation providing more evidence for the management of PH in the rat model. Moreover, lung ultrasound provided a noninvasive method in monitoring the establishment of animal models for basic and clinical studies of PH.

## 1. Introduction

Pulmonary hypertension (PH) was a deadly disease of the respiratory and circulatory systems, characterized by pulmonary vasoconstriction, pulmonary vascular remodeling, and in-situ thrombosis, all of which caused a progressive elevation in pulmonary vascular resistance, and ultimately, right heart failure, and death [1, 2]. PH is caused by a variety of etiologies. The main pathological features of PH were enhanced pulmonary arterial systolic reactivity and reconstruction of pulmonary arterial vascular anatomy. At present, the common treatment method for PH is drug therapy. Although some symptoms, activity endurance and survival rate of patients, can be significantly improved after treatment, the long-term survival rate is still low. The treatment of PH is still a major difficulty in clinical treatment, and it is also one of the focuses in medical circles. PH animal models play an irreplaceable role in revealing its pathophysiological mechanism and targeted drug development. The success of the PH rat model is mainly determined by the measurement of pulmonary artery pressure. Both traditional pulmonary artery pressure measurements adopt the right heart catheter method, and our team improved transthoracic ultrasound-guided puncture measurement technology which was easy for causing hemorrhage or death and failure of pressure measurement in rats [3]. Hence, it is necessary to consider the cost and time of establishing the PH model in rats. Experimental animal ethics requires the three principles of substitution, reduction, and optimization in medical research to ensure the welfare of experimental animals [4]. A better reference to judge the successful establishing of PH animal models and reduce animals' trauma and unnecessary death is urgently needed.

For many years, lung ultrasound (LUS) was limited to the study of pleural effusions and superficial pleural masses because alveolar air and bone limited the transmission of the ultrasound beam. In 1997, Lichtenstein's team first described the relationship between alveolar interstitial syndrome and the “comet tail” sign (line B) of the lung, thus pioneering the clinical application of LUS [5]. In recent years, it has been found that lung ultrasound was very sensitive to changes in lung gas content and the balance between gas and liquid. Subsequently, LUS has been widely used in the diagnosis and differential diagnosis of a variety of lung diseases, especially in the emergency scene where medical conditions were limited and in the intensive care unit where patients' movement were significantly limited, such as emergency departments, medical wards, and critical care units. Studies both in vivo and in vitro demonstrate that the number and type of artifacts visualized change with lung density [6]. This has led to the idea of a quantitative lung ultrasound approach, opening up new prospects for use not only as a diagnostic but also as a monitoring tool. With the continuous deepening of clinical research, lung ultrasound had been reassessed and become an important method to evaluate acute lung injury, ARDS, and other lung lesions at present [7, 8]. In addition, pulmonary ultrasonography demonstrated greater sensitivity and specificity for pleural effusion detection compared to clinical examination and chest radiograph [9]. Numerous clinical investigations have shown that there was a strong correlation between the number of B-lines in lung ultrasonography imaging and the level of pulmonary edema [10]. However, pulmonary ultrasound is not a true image of an organ and therefore has many limitations in terms of quantitative assessment of disease. There were few studies on the application of lung ultrasound in PH.

In this study, a PH rat model was induced by MCT. The ultrasound and pathological results were analyzed with a semiquantitative score. The primary endpoint of the study was the pathological score, and the secondary endpoint of the study was mean pulmonary artery pressure (mPAH). The correlation between lung ultrasound and pathology was evaluated to provide a theoretical basis for lung ultrasound of PH. Moreover, we had to provide a noninvasive method in monitoring the establishment of animal models for basic and clinical studies of PH.

## 2. Materials and Methods

### 2.1. Animal Grouping and Administration

**Figure 1:**
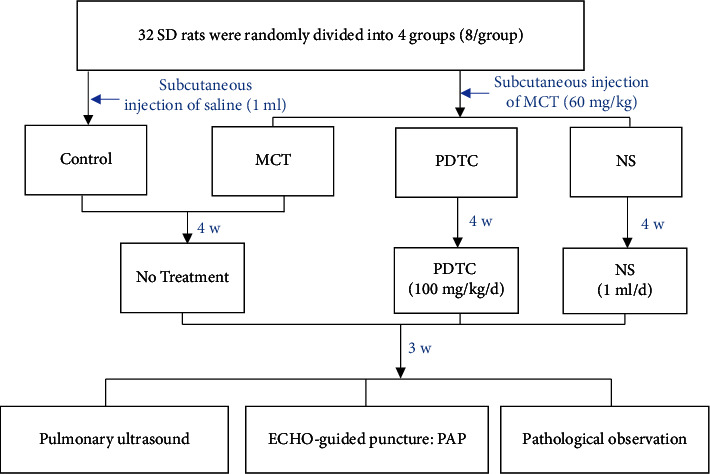
Flow chart of grouping and administration of rats in each group. Abbreviations: MCT, monocrotaline; PDTC, pyrrolidine dithiocarbamate; NS, normal saline; ECHO, echocardiography; PAP, pulmonary artery pressure.

This study established the PH rat model by monocrotaline (Beijing Solarbio Science & Technology, USA) and monitored it for 4 weeks. Then, we proposed administering PDTC (Beijing Solarbio Science & Technology, USA) therapy after MCT injection and monitored it for 3 weeks. 8-week-old male Sprague–Dawley (SD) rats were provided by the Animal Experiment Center of Guangxi Medical University (license number: SCXK GUI 2022–0003). All animal procedures and protocols were approved by the Experimental Animal Ethics Committee of Guangxi Medical University (ethic committee authorization number: NO.2023-D086-01). Totally 32 SD rats were randomly divided into the control group (*n* = 8), MCT group (*n* = 8), PDTC group (*n* = 8), and NS group (*n* = 8). The MCT group, PDTC group, and NS group received a single subcutaneous injection of MCT (60 mg/kg), while the control group received an equivalent volume of saline (1 ml). After the end of models, the PDTC group and the NS group received PDTC (100 mg/kg/d) and NS (1 ml) daily for treatment (Figure 1).

### 2.2. Pulmonary Ultrasound

According to the current recommendations, pulmonary ultrasonography measurements were carried out [11].

**Figure 2:**
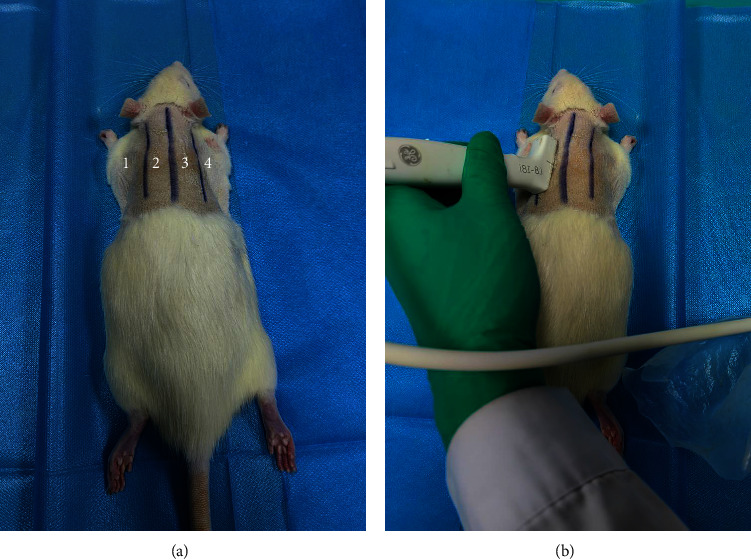
(a) Zoning of the rat chest wall. The examined rat was placed in the prone position, and pulmonary ultrasound consisted of bilateral scanning of the posterior (dorsal) and lateral chest walls. Each hemithorax was divided into two zones. A total of four scans were performed for each rat. 1: from the posterior axillary line to the scapular line. 2: from the scapular line to the paravertebral line. 3: from the paravertebral line to the scapular line. 4: from the scapular line to the posterior axillary line. (b) The probe positioning was used for the LUS acquisition.

**Figure 3:**
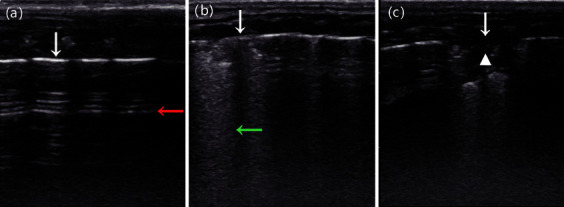
(a) Lung ultrasound score 0, the pleural line (white arrow) and A-line (red arrow) are clear. (b) Lung ultrasound score 1, the pleural line (white arrow) and A-line are blurred or disappeared, and B-line (green arrow) can be seen. (c) Lung ultrasound score 2, the pleural line (white arrow) disappeared with lung consolidation and air bronchogram (white triangle area).

The rats were anesthetized with 2% sodium pentobarbital intraperitoneally before examination, and the examined rat's back was shaved in order to cover the probe with gel. The rat was positioned in the prone position, and the pulmonary ultrasonography involved bilateral scanning of the dorsal and lateral chest walls for each hemithorax: (1) from the posterior axillary line to the scapular line and (2) from the scapular line to the paravertebral line (Figure 2). A total of four scans were performed for each rat. Each area was scored with the most severe ultrasound signs and recorded results according to the literature for ultrasound diagnosis of lung injury in rats (Figure 3) [12]. Score 0: lung pleural lines and A lines clear, without or only a few B lines, without air bronchogram, lung consolidation change, and pleural effusion; score 1: lung pleural lines and A lines indistinct, B lines are greater than or equal to 3, scattered air bronchogram and lung consolidation change, without pleural effusion; score 2: lung pleural lines and A lines vanish, B lines were dense and unevenly distributed, air bronchogram and lung consolidation change were obvious, extended and involved deep tissues, with or without pleural effusion. The lung ultrasound score of the rat was the sum of each area. All images were evaluated by two physicians with more than 5 years of experience in ultrasound diagnosis. Neither the rats' details nor the pathological results were available to the physician.

### 2.3. Pulmonary Artery Pressure Measurement

The surgeon gently placed the puncture needle into the right ventricular outflow system in the parasternal short-axis view under the supervision of real-time echocardiography. The puncture needle was gently advanced to the major pulmonary artery and recorded the pulmonary artery pressure curve when the right ventricular pressure curve emerged on the BL-420F biosignal acquisition and analysis system (Chengdu Taimeng Software Co., Ltd.)

### 2.4. Pathological Observation

Before the rats were sacrificed under excessive anesthesia, heparin (10U/100 g) was injected through the tail vein to prevent blood coagulation. Lung tissue was removed quickly and cleaned with normal saline and then observed in general. In SD rats, the blood flow rate of the main pulmonary artery was about 0.5 m/s. We injected saline steadily and slowly (<0.5 m/s) from the RV to clean the pulmonary vessels. Once the saline was clean, the entire lung tissue and other tissues were fixed in 10% formalin solution for 48 h, dehydrated with alcohol, transparent with xylene, embedded in paraffin, and sliced. Sections were stained with hematoxylin and eosin (H&E). At high magnification (×200), tissues of lung were observed for signs of endothelial cells injury, smooth muscle cells hyperplasia, inflammation, pulmonary arteriole wall thickness, and whether there was stenosis in the vessel cavity. 5 horizons were randomly observed, and the pathological score was performed for lung injury (Table 1). The pathology score of lung tissue was the sum of the 5 horizons. All images were evaluated by two pathologists with more than 5 years of experience in pathological diagnosis. Neither the rats' details nor the lung ultrasound results were available to the pathologist.

### 2.5. Statistical Analysis

The statistical analysis was performed using SPSS version 26.0 (IBM, USA), Statistical Product and Service Solutions software. One-way ANOVA was used to examine data that had a normal distribution and homogeneous variance; otherwise, the Kruskal–Wallis test was used. Correlation analysis was performed using Pearson correlation. *p* < 0.05 was considered statistically significant. An extremely strong positive correlation was defined as *r* > 0.7.

## 3. Results

### 3.1. Characteristics of Rats in Each Group

Rat mortality rates in each group ranged from 12.5 to 25% during the modeling phase. Lung ultrasonography was used to monitor the survivors in each group, and PAP measurements were performed effectively with the aid of echocardiography. The HR was 366–433 times/min in rats, which was in the normal rat heart rate range. Therefore, the result indicated that pentobarbital had no significant effect on the cardiovascular system and could not impact the measurement of PAP (Table 2).

### 3.2. Lung Ultrasound Score of Rats in Each Group

**Figure 4:**
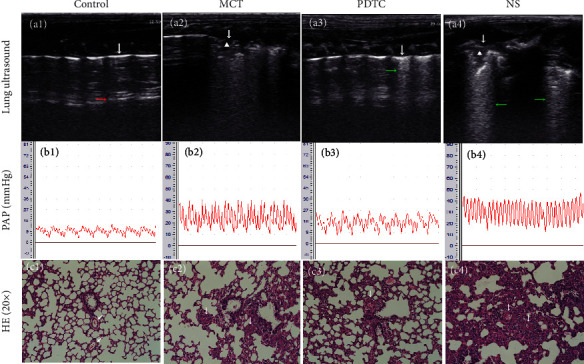
(a) Lung ultrasound features in each group, a1: the pleural line (white arrow) and A-line (red arrow) are clear; a2: the pleural line disappeared with lung consolidation and air bronchogram (white triangle area); a3: the pleural line and A-line are blurred, and B-line (green arrow) still can be seen; a4: the pleural line disappeared, and B-line increased with lung consolidation and air bronchogram. (b) PAP pressure curve in each group. (c) H&E staining of lung tissues, c1: a few inflammatory cell infiltration, pulmonary arteriole wall, and vascular cavity were normal without smooth muscle cell proliferation; c2: smooth muscle cell proliferation, pulmonary arteriole wall thickening, vascular cavity stenosis, with a large number of inflammatory cell infiltration; c3: compared with the MCT group, the pulmonary arteriole wall was thinner and the vascular cavity was enlarged with inflammatory cell infiltration decreased; c4: smooth muscle cell proliferation obvious, pulmonary arteriole wall thickening, vascular cavity stenosis even occlusion with inflammatory cell infiltration. Abbreviations: PAP, pulmonary artery pressure; MCT, monocrotaline; PDTC, pyrrolidine dithiocarbamate; NS, normal saline.

**Figure 5:**
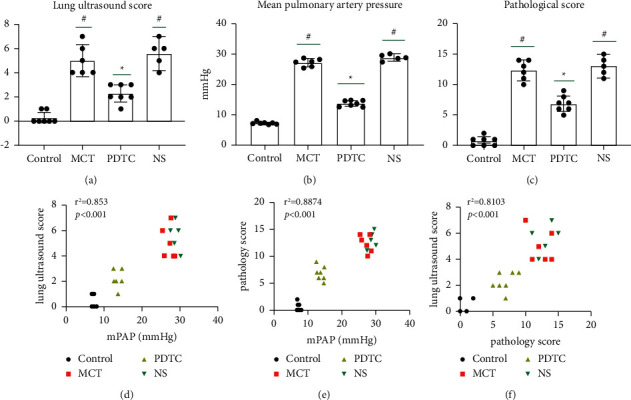
(a) Lung ultrasound score in each group. (b) Mean pulmonary pressure in each group. (c) Pathological score in each group. (d) Lung ultrasound score was positively correlated with mean pulmonary artery pressure (mPAP). (e) Pathological score was positively correlated with mPAP. (f) Lung ultrasound scores were positively correlated with pathological score.

The sum score of pulmonary ultrasound and differences between each group are displayed in Table 3. (1) The control group showed smooth, clear, and regular pleural line with parallel A lines, and there was no pleural effusion or pulmonary interstitial edema. MCT groups displayed blurred, unregular pleural line and A-line disappeared; B-line can be seen with lung consolidation and air bronchogram. The MCT group had increased pulmonary ultrasound score compared with the control group (*p* < 0.05). Compared with the MCT group and the NS group, the number of B-lines decreased, lung consolidation and pleural effusion were rare in the PDTC group rats after treatment, while solid lung changes are common in the NS group for lung ultrasound (Figure 4(a)). The pulmonary ultrasound score decreased in the PDTC group compared with the MCT group and NS group (*p* < 0.05), while there was no significant difference in the pulmonary ultrasound score between the MCT group and the NS group (*p* > 0.05) (Figure 5(a)).

### 3.3. Pulmonary Artery Pressure of Rats in Each Group

The results are presented in Table 3 and Figures 4(b) and 5(b). Pulmonary artery pressure (PAP) increased in the MCT group, PDTC group, and NS group. Then, PAP was reduced in the PDTC group, while the NS group still increased after treatment. There was statistical difference between the control group and the MCT group (*p* < 0.05) which mean that the rat model of PH was established successfully. Compared with the MCT group and the NS group, PAP was decreased in the PDTC group (*p* < 0.05), while there was no significant difference in PAP between the MCT group and the NS group (*p* > 0.05), which mean that PDTC could reduce the damage of pulmonary vascular endothelial cells and effectively delay the progression of PH and had certain therapeutic effect.

### 3.4. Lung Tissue Pathological Score of Rats in Each Group

The sum pathological score of lung tissue and differences between each group are displayed in Table 3. Compared with the control group, the lung histomorphology of the MCT group showed significant changes, pulmonary vascular endothelial necrosis, smooth muscle cell proliferation, pulmonary arteriole wall thickening, and vascular cavity stenosis, with a large number of inflammatory cell infiltration. The MCT group had increased the pathological ultrasound score compared with the control group (*p* < 0.05). The pulmonary arteriole wall was thinner, and the vascular cavity was enlarged with the number of inflammatory cell infiltration decreased, while the lung histomorphology had no significant change or was even severe in the NS group compared with the MCT group after treatment (Figure 4(c)). There was no significant difference in the pathological score between the MCT group and the NS group (*p* > 0.05). The pathological score decreased in the PDTC group compared with the MCT group and the NS group (*p* < 0.05) (Figure 5(c)).

### 3.5. Correlation Analysis

The results are presented in Figures 5(d), 5(e), and 5(f). We investigated both lung ultrasound score and the pathological score were positively correlated with mean pulmonary artery pressure (mPAP) (both *r* > 0.8, *p* < 0.0001). Moreover, lung ultrasound scores were positively correlated with pathological score (*r* > 0.8, *p* < 0.0001).

## 4. Discussion

Normal lung is gas-containing tissue, and there is a large acoustic impedance difference between it and the soft tissue on the body surface. In the process of ultrasound transmission, the gas-containing organ will lead to strong reflection and large echo and present as bright spot, strong blob echo, which can be shown as a bright, clear, and regular pleural line near the pleura. The mainly pathological feature of pulmonary hypertension caused by monocrotaline (MCT) was that monocrotaline damages pulmonary vascular endothelial cells and makes pulmonary vascular constriction after liver metabolism. Meanwhile, by releasing inflammatory cytokines and chemokines, the damaged endothelial cells gather a large number of inflammatory cells in the blood vessels, causing abnormal proliferation of blood vessel wall cells, thickening of vessel wall, and narrowing of vessel cavity, eventually leading to increased pulmonary vascular resistance and pulmonary artery pressure [13, 14]. Due to the effect of injurious factors, pulmonary capillary endothelial cells and alveolar epithelial cells damaged, pulmonary capillary permeability and alveolar exudation increased, and the composition of lung surfactant changed, resulting in pulmonary interstitial edema, alveolar collapse, reduction in lung tissue gas content, and changes in lung tissue structure and tissue elasticity. As a result, ultrasonic characteristics of the diseased lung tissue such as acoustic conduction and echo intensity changed.

Our study indicated that ultrasonography was sensitive to the changes of pulmonary tissue signs in PH rats. In this study, a rat model of pulmonary hypertension was induced by intraperitoneal injection of MCT. The histopathological results were consistent with the pathological characteristics of pulmonary hypertension. Compared with the control group, the lung histomorphology of the MCT group showed significant changes, pulmonary vascular endothelial necrosis, smooth muscle cell proliferation, pulmonary arteriole wall thickening, and vascular cavity stenosis, with a large number of inflammatory cell infiltration. The results are consistent with related studies [15, 16]. The pulmonary arteriole wall was thinner, and the vascular cavity was enlarged with the number of inflammatory cell infiltration decreased after PDTC treatment.

In this study, we found that the lung ultrasound findings were sensitive to the changes of pulmonary tissue signs in PH rats. The ultrasonic manifestations of the MCT group were the pleural line, and the A-line disappeared, the number of B-lines increased, B-line fusion was observed, and lung consolidation and bronchial signs were observed. The lung ultrasound scan was consistent with the pathology of PH caused by MCT. Endothelial cell injury increased capillaries' permeability resulting in pulmonary edema. On the other hand, injury of pulmonary vascular endothelial cells leads to inflammatory response, bleeding, and thrombosis which promotes the contraction of vascular smooth muscle cells, resulting in strong contraction of pulmonary vessels, and the pulmonary vascular resistance increased, causing PH. PH further promoted pulmonary interstitial edema [17, 18]. After PDTC treatment, lung ultrasound showed obvious changes compared with the MCT group and the NS group, mainly manifested in the number of B-lines decreased, lung consolidation was rare, and the LUS sum score improved. Correlation analysis presented that both the lung ultrasound score and the pathological score were positively correlated with mean pulmonary artery pressure (mPAP). Moreover, lung ultrasound scores were positively correlated with the pathological score.

Studies had shown that lung ultrasound can monitor changes in the lungs, assess alveolar and pulmonary interstitial damage, and provide a semiquantitative basis for an extravascular pulmonary water index. It is also believed that the presence of more than 3 B-lines in the lung field indicates the presence of alveolar interstitial syndrome [19, 20]. This study also confirmed that lung ultrasonography was sensitive to pulmonary interstitial edema and increased alveolar exudation and collapse. Therefore, we believed that lung ultrasound had great correlation with pulmonary pathological manifestations and could play an important role in monitoring the progression and efficacy of PH in the rat model.

As an emerging test method, lung ultrasound was very popular in recent years which offered great convenience in diagnosing and managing diseases. However, each imaging method has its limitations: (1) pulmonary ultrasound can only detect subpleural lesions; (2) “different diseases with the same manifestations” phenomenon is common which needs careful identification, such as B-lines can be associated to different pathological patterns [21]; and (3) quantitative assessment of lung ultrasound remains difficult. More in-depth research is urgently needed.

## 5. Conclusion

Ultrasonography was positively correlated with the pathological and could effectively evaluate the severity of PH in rats. Lung ultrasound evaluation in PH rats provided more evidence for the management of PH in the rat model. Moreover, lung ultrasound provided a noninvasive method in monitoring the establishment of animal models for basic and clinical studies of PH.

## Figures and Tables

**Table 1:** Lung tissue pathological score criteria of PH induced by MCT.

Index	0	1	2	3
Endothelial cells	Normal	Swelling	Partial necrosis	Almost necrosis
Smooth muscle cells	None	Mild hyperplasia	Moderate hyperplasia	Severe hyperplasia
Inflammatory cells	None	Mild infiltration	Moderate infiltration	Extensive infiltration
MT%	<30%	30%–40%	40%–50%	>50%
VA%	>45%	30%–45%	25%–30%	<25%

Abbreviations: PH, pulmonary hypertension; MCT, monocrotaline; MT: index of pulmonary arteriole wall membrane thickness (2 × MT/ED, ED: external diameter of pulmonary arteriole); VA%: index of vascular cavity area (VA/TAA, TAA: total vascular area).

**Table 2:** Characteristics of rats in each group.

Group	Case (*n*)	Death (*n*)			Survival (*n*)
		Anesthesia accident	Right heart failure	Lung lesions	
Control group	8	1	0	0	7
MCT group	8	1	1	0	6
PDTC group	8	1	0	0	7
NS group	8	1	1	1	5

Abbreviations: MCT, monocrotaline; PDTC, pyrrolidine dithiocarbamate; NS, normal saline.

**Table 3:** Lung ultrasound score, PAP, and lung tissue pathological score in each group.

Group	Case (*n*)	Ultrasound score	mPAP (mmHg)	Pathological score
Control group	7	0.29 ± 0.48	7.23 ± 0.43	0.71 ± 0.76
MCT group	6	5.00 ± 1.27^▲^	27.22 ± 1.31^▲^	12.33 ± 1.63^▲^
PDTC group	7	2.29 ± 0.76^★◆^	13.73 ± 0.10^★◆^	6.86 ± 1.35^★◆^
NS group	5	5.60 ± 1.14^▲^	28.92 ± 1.58^▲^	13.00 ± 1.58^▲^

Abbreviations: mPAP, mean pulmonary artery pressure; MCT, monocrotaline; PDTC, pyrrolidine dithiocarbamate; NS, normal saline; ^▲^: compared with the control group, there was significant difference (*p* < 0.05); ^★^: compared with the MCT group, there was significant difference (*p* < 0.05); ^◆^: compared with the MCT group, there was significant difference (*p* < 0.05).

## Data Availability

All data relevant to the study are included in the article and are available from the corresponding author on reasonable request.

## Supplementary Materials

Supplementary Materials Each parameter for single animals.
