# Supplementary material for: The Correlation between Lung Ultrasound and Pathology in Rat Model of Monocrotaline-Induced Pulmonary Hypertension
Source: Can Respir J. 2024 Jul 23;2024:6619471. doi: 10.1155/2024/6619471 (PMC11288697; doi:10.1155/2024/6619471)
Supplement: Supplementary Materials — Each parameter for single animals. [file 6619471.f1.pdf]

lung score	control	MCT	PDTC	NS
	0	4	3	6
	0	4	3	5
	1	5	2	6
	1	7	2	4
	0	4	2	7
	0	6	3	
	0		1	
Spap(mmHg)	14.3	37.3	24.1	42.3
	12.4	38.5	23.2	43.6
	13.6	41.3	24.6	40.5
	12.6	40.1	22.8	39.4
	13.8	39.5	23.7	45.8
	12.3	40.6	21.3	
	12.1		24.8	
mPAP（mmHg）	8.1	25.9	12.5	29.6
	7.4	28.3	14.8	28.6
	6.9	27.4	13.2	27.5
	7.3	27.6	12.6	30.1
	7.2	28.7	14.7	28.8
	7.1	25.4	14.6	
	6.8		13.7	
Dpap（mmHg）	2.5	14.3	4.3	15.1
	2.1	12.9	3.8	16.3
	3.1	14.6	3.6	15.9
	2.6	15.1	4.5	14.6
	2.2	14.7	5	16.1
	2.3	15.6	4.2	
	2.7		3.7	
Pathological score	0	13	9	15
	1	14	8	13
	2	12	6	11
	0	10	7	12
	1	11	5	14
	1	14	6	
	0		7	
HR（times/min）	428	379	371	418
	401	413	388	421
	375	377	396	416
	412	411	372	433
	366	422	410	400
	404	396	381	
	382		408	
